# In memoriam: Dr Mohamed F. Sukkarieh (1952–2021)

**DOI:** 10.1080/2090598X.2021.2004036

**Published:** 2021-12-01

**Authors:** Mohamad Moussa, Mohamad Abou Chakra

**Affiliations:** Chairman of the Urology Department, Zahraa Hospital, University Medical Center, Lebanese University, Beirut, Lebanon; Faculty of Medicine, Urology Department, Lebanese University, Beirut, Lebanon mohamedabouchakra@hotmail.com

Sunday morning, 12 September 2021, Mohamed F. Sukkarieh passed away aged 69 years due to complications from coronavirus disease 2019 (COVID-19) infection. He left this life the way he lived it, in the difficult lane. His name deserves to be remembered and honoured.

He was born in 1952 in Beirut, Lebanon. He entered the Faculty of Medicine of Damascus in Syria, then he joined the residency programme in surgery and urology at the Rostock University in Germany. He returned to Lebanon during the civil war in 1985, where he was appointed as a urologist at Berbir hospital. He encountered several difficulties during that time due to the unstable situations in Lebanon and the gruesome war. He did not give up and emigrated as did many physicians in that period, he insisted on supporting his patients in difficult times. Afterward, Dr Sukkarieh worked in the Middle East hospital, Sahel General Hospital, and Al Zahraa Hospitals, serving the committee with sincerity. We all know Dr Sukkarieh as the ‘father of the poor’.

During that time, Dr Sukkarieh always thought about his Arab colleagues, he always said ‘I hope to see a moment when all Arab urologists get their association and their regular urology meeting’. He co-founded the Arab Association of Urology (AAU), and he was appointed as a treasurer of the AAU between 1999 and 2003. In late 2001, he co-founded the *Arab Journal of Urology* (AJU), which is the official journal of the AAU. The *AJU* publishes high-standard peer-reviewed research on all aspects of urology to the widest possible urological community worldwide. Dr Sukkarieh was always present in all scientific meetings locally and regionally, discussing the novelty of therapy in all urological fields. He organised many conferences in Lebanon in the late 1990s, comprising urological oncology, paediatrics, infertility, and endourology topics. His achievements could not have been possible without the active involvement of all colleagues from Arab countries.

Dr Sukkarieh possessed immense humanity, a deep vocation for service, and a generous attitude towards life. His presence in our lives is sorely missed by us all.

